# Breast cancer survivors' risk of interval cancers and false positive results in organized mammography screening

**DOI:** 10.1002/cam4.3182

**Published:** 2020-06-30

**Authors:** Sisse Helle Njor, Ilse Vejborg, Mette Bach Larsen

**Affiliations:** ^1^ Department of Public Health Programmes Regional Hospital Randers Randers Denmark; ^2^ Department of Clinical Medicine Aarhus University aarhus Denmark; ^3^ Department of Radiology Copenhagen University Hospital Rigshospitalet Denmark

**Keywords:** cancer screening, cancer survivors, false negative reactions, false positive reactions, mammography

## Abstract

**Background:**

Breast cancer survivors are increasing followed for new breast cancers / recurrences by mammography screening only. We aimed at assessing how often breast cancer survivors get a false positive or false negative result at mammography screening.

**Methods:**

All mammography screenings performed between 2007 and 2017 in the Danish national mammography screening programme were included. Screenings in women with a breast cancer diagnosis prior to invitation were included in the “breast cancer survivors” group, while remaining screenings were included in the “no previous breast cancer” group. We compared the proportion of false positive screenings and the proportion of breast cancers detected at screening among breast cancer survivors and women without previous breast cancer. The analyses were further stratified according to whether the women had a diagnostic breast imaging in the 21 months prior to the screening.

**Results:**

At initial screenings, breast cancer survivors had a significant lower false positive risk than other women, while the risk was similar at subsequent screenings. Breast cancer survivors had a significant lower proportion of breast cancers detected at screening compared to other women. This was true both for women who had a diagnostic breast imaging in the 21 months prior to screening and those who did not.

**Conclusion:**

This study shows that breast cancers survivors have a smaller amount of their new breast cancers detected at mammography screening, when compare to the amount of new breast cancers detected at mammography screening among women without previous breast cancer. The lower sensitivity does not seem to be due to different behavior among breast cancer survivors.

## Graphical abstract

Proportion of breast cancers detected at mammography screening among women with and without previous breast cancer screened at initial and subsequent screening.

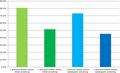

**MESSAGE**

- Breast cancer survivors have a similar or slightly lower risk of a false positive mammography screening than other women.
- Breast cancer survivors have 30%‐40% less of their recurrences and new breast cancers discovered at mammography screening than other women.

## 1 INTRODUCTION

For breast cancer survivors, the annual hazard of a recurrence is about 10% during the first 5 years and remains at 2%‐5% for the following 15 years. The hazard is not negligible for at least 24 years.^1^, ^2^ Given the high risks of recurrences, a high‐quality follow‐up programme for breast cancer survivors is essential to decrease breast cancer morbidity and mortality.

In a paper from 2013 ESO‐MBC (European School of Oncology – Metastatic Breast Cancer) Task Force recommended against intensive, routine radiologic or blood‐based surveillance (with the exception of mammography) in breast cancer survivors. Although they also stated that “as systemic therapies for MBC continue to improve, this question might be re‐visited”.^3^

It is known that mammography screening decreases breast cancer mortality in a standard population, by finding breast cancers at an earlier stage.^4^, ^5^ It therefore seems likely that mammography screening will also detect new cases and recurrences in breast cancer survivors at an earlier stage and thereby decrease breast cancer mortality.

Several reviews in which there was a high level of overlapping studies, have found that there is better survival among recurrences detected at mammography than among recurrences detected through other methods.^6^, ^7^, ^8^ In their 2016 review Muradali et al concluded that "organized screening programs should reassess their guidelines on surveillance mammography and consider including breast cancer survivors".^8^ All reviews though state that further evidence is needed, since better survival is biased by length and lead time bias.

Difficulties in interpreting mammograms in breast cancer survivors might affect the quality of mammography screening and thereby decrease the benefit for breast cancer survivors,^9^, ^10^, ^11^, ^12^ wherefore it is important to monitor how large a proportion of recurrences is and new breast cancers that are found at mammography screening. There are very little data on this among breast cancer survivors and none of these included all breast cancer survivors.

It seems likely that the negative psychological impact from a false positive mammography^13^, ^14^ is higher among previous breast cancer patients than among women without a previous breast cancer. Therefore, amongst previously breast cancer operated women it is even more necessary than in the general screening population to keep the percentage of false positive as low as possible but still keeping a high detection rate.

This study estimates how often breast cancer survivors attending mammography screening receive a false positive results and how often their recurrences and new breast cancers are detected at mammography screening. To investigate the impact of previous diagnostic breast imagings, we also stratified by whether the women recently had a diagnostic breast imaging or not.

## 2 MATERIAL AND METHODS

### 2.1 Setting

In February 2015 the national board of health in Denmark published a new follow‐up programme for breast cancer, stating that breast cancer survivors aged 50‐79 years who had mastectomy should have biennial mammography screening as the only routine follow‐up. Breast cancer survivors aged 50‐79 years who had lumpectomy should have a diagnostic mammography including breast palpation and ultrasound examination 18 months after the operation and thereafter biennial mammography screening as the only follow‐up unless less being the carrier of a risk increasing gene mutation.^15^, ^16^ Prior to 2015, the Danish guidelines from 2004, 2010 and 2012 recommended that breast cancer survivors had a clinical breast examination at least every 6 months for 5 years and thereafter every 12 months for 5 years.^17^, ^18^, ^19^ The 2012 guidelines recommended that low‐risk patients could stop being followed with clinical breast examinations after 3 years. The 2004 and 2010 guidelines recommended that breast cancer survivors had mammography and eventual ultrasound every second year, although the 2004 guidelines recommended annual mammography to all breast cancer survivors who had lumpectomy. In the 2012 guidelines breast cancer survivors who had mastectomy were recommended to have biennial mammography screening whereas breast cancer survivors who had lumpectomy were recommended a diagnostic mammography 18 months after the operation, followed by biennial mammography, and eventually mammography screening.

Nationwide mammography screening started in 2007 in Denmark, with full coverage by 2010. All women aged 50‐69 are invited every second year to mammography screening, unless the women who actively opted out. Screening and subsequent assessment and treatment are offered free of charge. The screening programme is managed by the five Danish regions according to the national guidelines^20^ and the quality of the program is monitored in the Danish Quality Database for Mammography Screening.^21^ Double reading were used in all regions and no region used Computer Assisted Detection systems.^22^

### 2.2 Study population

This study included all mammography screening examinations from the Danish national mammography screening programme, performed from the start of the national mammography screening programme in 2007 until 31/12‐2014. Screening mammographies in women who had a breast cancer diagnosis at least a year before the invitation to this screening were included in the group “breast cancer survivors” whereas the remaining screenings were included in the group of “no previous breast cancer”.

### 2.3 Data

Data on previous breast cancers were retrieved from the Danish National Cancer Register. The register includes data on cancers from 1943 and has been shown to have a high degree of completeness.^23^ Information on invitations, participation, whether the screening was positive or negative, number of screen‐detected cases, and number of interval cancers were retrieved from the Danish Quality Database for Mammography Screening.^24^ The Danish Quality Database for Mammography Screening retrieved the data on participation, whether the screening was positive or negative, number of screen‐detected cases and number of interval cancers from the Danish National Patient Register, and the Danish National Pathology Register.^25^, ^26^ The registers were linked using the Danish personal identification numbers (CPR numbers). Information about diagnostic breast imagings were retrieved from the Danish National Patient Register and included the following procedures: Diagnostic mammography, Diagnostic Mamma tomosynthesis, ultrasound investigation of mamma, MR scanning of mamma, Diagnostic MR scanning of mamma and Needle biopsy of breast.

### 2.4 Definitions

A screen‐detected case was defined as an invasive cancer or ductal carcinoma in situ that was diagnosed within 6 months of a positive screening. An interval cancer was defined as an invasive cancer that was diagnosed between a negative screening and the next screening/ 2 years (whatever came first) or diagnosed more than 6 months after a positive screening but before the next screening/ 2 years (whatever came first). A false positive test was defined as a positive screening that did not result in a screen‐detected case.

### 2.5 Analyses

We compared the proportion of false positive screenings among breast cancer survivors and women with no previous breast cancer.

All breast cancers diagnosed in breast cancer survivors, ie, both recurrences and new breast cancers were included in the analyses. Breast cancer survivors have a larger risk of getting a breast cancer than women who have never had a breast cancer diagnosis before. Due to this, the detection rate (number of breast cancers detected at mammography screening per screened woman) as well as the interval cancer rate (number of interval cancers per screened woman) might be higher among breast cancer survivors than among the general population, even though mammography screening is as efficient for breast cancer survivors as for the general population. A comparison of these numbers will therefore not necessarily tell whether breast cancer screening is as beneficial to breast cancer survivors as to the general population. The proportion of screen‐detected cancers and interval cancers that are detected at screening give an estimate of the sensitivity ie, the odds of having the breast cancer detected at screening given that you have breast cancer. A low sensitivity tells that many breast cancers are not found at screening, ie, the screening is not that efficient or a large amount of the cancers are very fast growing. Sensitivity is not affected by the total number of cancers in the population, wherefore we used this measure to compare whether mammography screening is as efficient in breast cancer survivors as in women without previous breast cancer diagnoses.

Women who are going for regular follow‐up outside the screening programme will have a clinical breast examination and/or a diagnostic mammography 0‐2 years after the screening. Breast cancers detected at this clinical breast examination or mammography will according to our definitions be classified as interval cancers, wherefore the sensitivity will be lower. We do not know if a woman is going for regular follow‐up or not, but we known if a women has had diagnostic mammography, diagnostic mamma tomosynthesis, ultrasound investigation of mamma, MR‐scanning of mamma, diagnostic MR‐scanning of mamma or needle biopsy of breast. As women going for regular follow‐up will very likely undergo at least one of these procedures during a period of 2 years, we used these procedures as a proxy for “regular follow‐up”. To avoid contamination with diagnostic breast imaging procedures at the previous mammography screening app 2 years ago, we only looked at breast imaging procedures during a period of 21 months prior to the mammography screening. We therefore included women who had at least one of these procedures 0‐21 months prior to the screening in a “with diagnostic breast imaging” group, whereas women without any of these procedures 0‐21 months prior to the screening were included in a “without diagnostic breast imaging” group. We stratified our analyses according to whether women had a diagnostic breast imaging in 21 months prior to the mammography screening, to investigate whether this influences the sensitivity.

## 3 RESULTS

We included 1 730 962 screenings of women with no previous breast cancer diagnoses and 31 078 screening of breast cancer survivors (Table 1). As expected due to an increased breast cancer risk among survivors, the breast cancer detection rate was higher among breast cancer survivors than among women with no previous breast cancer diagnoses. Breast cancer survivors were in general older and had a slightly higher proportion of their interval cancers detected in the first year after screening.

**Table 1:** Age, detection rate and number of interval cancers in first and second year for breast cancer survivors and women without previous breast cancer diagnoses

	No previous breast cancer diagnoses			Breast cancer survivors		
	÷ examination^1^ 0‐21 mo	+ examination^1^ 0‐21 mo	All	÷ examination^1^ 0‐21 mo	+ examination^1^ 0‐21 mo	All
Age (average)	59.5	58.1	59.5	62.9	61.7	62.7
Detection rate	0.70% (n = 1 705 402)	1.10% (n = 25 560)	0.71% (n = 1 730 962)	1.30% (n = 27 408)	1.12% (n = 3670)	1.28% (n = 31 078)
Interval cancer [0‐12[ mo after screening	1201 (34.1%)	66 (37.1%)	1267 (34.3%)	129 (39.9%)	45 (40.9%)	174 (40.1%)
Interval cancer [12‐24[mo after screening	2317 (65.9%)	112 (62.9%)	2429 (65.7%)	195 (60.2%)	65 (59.1%)	260 (59.9%)

^1^ Diagnostic breast imaging.

### 3.1 False positive risk

At initial screenings, women without previous breast cancer diagnoses had a false positive risk of 2.7%, whereas breast cancer survivors had a significant lower risk of 2.0% (Table 2). The false positive risk was quite similar for all groups of “years since last breast cancer diagnosis”. At subsequent screening, both women without previous breast cancer diagnoses and breast cancer survivors had a false positive risk of 1.6%. Again the false positive risk was quite similar for all groups of “years since last breast cancer diagnosis”.

**Table 2:** Proportions of screenings with a false positive result and relative risk (RR) of a false positive test for breast cancer survivors compared to women with no previous breast cancer according to years since last breast cancer diagnosis with 95% confidence intervals (CI)

	Initial mammography screening		Subsequent mammography screening	
	False positive proportion	RR (95% CI)	False positive proportion	RR (95% CI)
No previous breast cancer	2.7% (19231/719238)	1.00	1.6% (16671/1011784)	1.00
Years since last breast cancer diagnosis				
0‐1 y	2.1% (3/144)	0,78 (0.25‐2.39)	1.3% (2/153)	0.79 (0.20‐3.14)
1‐2 y	2.5% (16/628)	0,95 (0.59‐1.55)	1.9% (20/1051)	1.15 (0.82‐1.95)
2‐4 y	1.7% (19/1095)	0,65 (0.42‐1.01)	1.7% (42/2497)	1.02 (0.76‐1.38)
4‐6 y	1.4% (23/1654)	0,52 (0.35‐0.78)	1.5% (27/1797)	0.91 (0.63‐1.33)
6‐10 y	2.1% (73/3479)	0,78 (0.63‐0,99)	1.3% (29/2150)	0.82 (0.57‐1.18)
>10 y	2.1% (159/7398)	0,80 (0.69‐0.94)	1.7% (152/8972)	1.03 (0.88‐1.20)
All previous	2.0% (293/14398)	0.76 (0.68‐0.85)	1.6% (272/16620)	0.99 (0.88‐1.12)
>4 y	2.0% (255/12531)	0.76 (0.67‐0.86)	1.6% (208/12919)	0.98 (0.85‐1.12)

### 3.2 Sensitivity

Women without previous breast cancer diagnoses attending their initial screenings had 6040 cancers detected at mammography screening and 1434 interval cancers, giving a sensitivity of 80.8% (Table 3). Breast cancer survivors attending their initial screening had 182 cancers detected at mammography screening and 171 interval cancers, giving a sensitivity of 51.6%. The sensitivity was significantly lower among breast cancer survivors than among women without previous breast cancer diagnoses (RR: 0.64 [0.58‐0.71]). The sensitivity was significantly lower as long as 1 year had passed since the breast cancer diagnosis. Among women attending their subsequent screening the sensitivity was also significantly lower for breast cancer survivors compared to women without previous breast cancer diagnoses (RR: 0.62 [0.54‐0.68]). Again the sensitivity was significantly lower as long as 1 year had passed since the breast cancer diagnosis.

**Table 3:** Screen‐detected cancers out of all cancers (SD/(IC + SD)) and relative risk (RR) of getting a breast cancer detected at mammography screening for breast cancer survivors compared to women with no previous breast cancer according to years since last breast cancer diagnosis with 95% confidence intervals (CI)

	Initial mammography screening		Subsequent mammography screening	
	Proportion of screen‐detected cancers SD/(IC + SD))	RR (95% CI)	Proportion of screen‐detected cancers SD/(IC + SD))	RR (95% CI)
No previous breast cancer	80.8% (6040/7474)	1.00	73.2% (6172/8436)	1.00
Years since last breast cancer diagnosis				
0‐1 y	50.0% (7/14)	0.62 (0.37‐1.04)	57.9% (11/19)	0.79 (0.54‐1.16)
1‐2 y	8.3% (1/12)	0.10 (0.02‐0.67)	23.1% (6/26)	0.32 (0.16‐0.64)
2‐4 y	35.7% (10/28)	0.44 (0.27‐0.73)	30.8% (16/52)	0.42 (0.28‐0.63)
4‐6 y	36.0% (9/25)	0.45 (0.26‐0.75)	43.6% (17/39)	0.60 (0.42‐0.85)
6‐10 y	50.7% (36/71)	0.63 (0.50‐0.79)	38.7% (24/62)	0.53 (0.39‐0.72)
>10 y	58.6% (119/203)	0.73 (0.65‐0.81)	50.9% (142/279)	0.70 (0.62‐0.78)
All previous	51.6% (182/353)	0.64 (0.58‐0.71)	45.3% (216/477)	0.62 (0.54‐0.68)
>4 y	54.8% (164/299)	0.68 (0.61‐0.75)	48.2% (183/380)	0.66 (0.59‐0.73)

Among women with a diagnostic breast imaging in the last 21 months attending their initial screening, the sensitivity was 56.4% for women with no previous breast cancer and 33.3% for breast cancer survivors (Table 4). The sensitivity among breast cancer survivors was significantly lower than the sensitivity among women without a previous breast cancer (RR: 0.59 [0.43‐0.82]. When looking at subsequent screenings, the sensitivity was also significantly lower among breast cancer survivors than among women without a previous breast cancer (RR: 0.31 [0.19‐0.48].

**Table 4:** Screen‐detected cancers out of all (SD/(IC + SD)) among women with/without a diagnostic breast imaging in the last 21 months and relative risk (RR) of getting a breast cancer detected at mammography screening for breast cancer survivors compared to women with no previous breast cancer according to years since last breast cancer diagnosis with 95% confidence intervals (CI)

	Initial mammography screening		Subsequent mammography screening	
	Proportion of screen‐detected cancers SD/(IC + SD))	RR (95% CI)	Proportion of screen‐detected cancers SD/(IC + SD))	RR (95% CI)
*Diagnostic breast imaging in the last 21 months*				
No previous breast cancer	56.4% (124/220)	1.00	65.4% (157/240)	1.00
Years since last breast cancer diagnosis				
0‐1 y	36.4% (4/11)	0.65 (0.29‐1.42)	30.0% (3/10)	0.46 (0.18‐1.19)
1‐2 y	0.0% (0/9)	‐	18.2% (4/22)	0.28 (0.11‐0.68)
2‐4 y	15.4% (2/13)	0.27 (0.08‐0.98)	18.2% (2/11)	0.28 (0.08‐0.98)
4‐6 y	28.6% (2/7)	0.51 (0.16‐1.64)	25.0% (1/4)	0.38 (0.07‐2.10)
6‐10 y	64.3% (9/14)	1.14 (0.76‐1.71)	12.5 (1/8)	0.19 (0.03‐1.20)
>10 y	37.0% (10/27)	0.66 (0.40‐1.09)	21.4% (3/14)	0.33 (0.12‐0.90)
All previous	33.3% (27/81)	0.59 (0.43‐0.82)	20.3% (14/69)	0.31 (0.19‐0.48)
>4 y	43.8% (21/48)	0.78 (0.55‐1.09)	19.2% (5/26)	0.29 (0.19‐0.50)
*No diagnostic breast imaging in the last 21 months*				
No previous breast cancer	81.6% (5916/7254)	1.00	73.4% (6015/8196)	1.00
Years since last breast cancer diagnosis				
0‐1 y	100.0% (3/3)	‐	88.9% (8/9)	1.21 (0.96‐1.53)
1‐2 y	33.3% (1/3)	0.41 (0.08‐2.03)	50.0% (2/4)	0.68 (0.26‐1.82)
2‐4 y	53.3% (8/15)	0.65 (0.41‐1.05)	34.1% (14/41)	0.47 (0.30‐1.71)
4‐6 y	38.9% (7/18)	0.48 (0.27‐0.85)	45.7% (16/35)	0.62 (0.43‐0.90)
6‐10 y	47.4% (27/57)	0.58 (0.44‐0.76)	42.6% (23/54)	0.58 (0.43‐0.79)
>10 y	61.9% (109/176)	0.76 (0.68‐0.85)	52.5% (139/265)	0.71 (0.64‐0.80)
All previous	57.0% (155/272)	0.70 (0.63‐0.78)	49.5% (202/408)	0.67 (0.61‐0.74)
>4 y	57.0% (143/251)	0.70 (0.63‐0.78)	50.3% (178/354)	0.69 (0.62‐0.76)

Women who did not have a diagnostic breast imaging in the last 21 months had higher sensitivity both among women without a previous breast cancer as well as among breast cancer survivors (Table 4). As among women who had a diagnostic breast imaging in the last 21 months, the sensitivity was significantly lower for breast cancer survivors compared to women without previous breast cancer diagnoses.

## 4 DISCUSSION

This study showed that even though the detection rate is higher, breast cancer survivors had 30%‐40% less of their new breast cancers detected by mammography screening compared to women without previous breast cancer diagnoses. Whereas out of 81% breast cancers found at initial mammography screening of women without a previous breast cancer only 52% of breast cancers were found at initial mammography screening of breast cancer survivors. At subsequent screening 73% of new breast cancers were detected at mammography screening of women without previous breast cancer diagnoses and 45% of breast cancers were detected at mammography screening of breast cancer survivors. A significant difference was found both for women who had a diagnostic breast imaging 0‐21 months prior to mammography screening as well as for women without such a diagnostic breast imaging.

### 4.1 Strengths and weaknesses

The major strength of this study is the large number of breast cancer survivors and screenings, as well as the fact that all information was obtained taken from high‐quality registers.

Interval cancers are defined as invasive cancers that were diagnosed from 0 to 24 months after a negative screening or diagnosed 6‐24 months after a positive screening. Screen‐detected cases were defined as invasive cancers and ductal carcinoma in situ that was diagnosed within 6 months of a positive screening. If the assessment after a positive screening takes close to or more than 6 months, this definition could have led to screen‐detected cases being diagnosed as interval cancers. Similarly interval cancers occurring quickly after a false positive test can lead to interval cancers being diagnosed as screen‐detected cancers. To quantify this problem, we compared data from 2008 to 2011 in one of the Danish regions (The Capital Region) with data from DKMS (data used in this study). The data from the Capital Region included information on screen‐detected cases as well as interval cancers provided by the radiologists. This comparison revealed that among 1619 interval cancers three interval cancers might have been falsely defined as screen‐detected in DKMS. Among 2143 screen‐detected cancers one was probably falsely defined as interval cancer in DKMS. These very low numbers of misclassification may not have affected our results.

All women were followed for interval cancer 24 months after the screening unless they died or emigrated in this period. Given that breast cancer survivors have a higher risk of dying than women without a previous breast cancer diagnosis the follow‐up time for breast cancer survivors is slightly lower than that of the follow‐up time for women without a previous breast cancer diagnosis. The difference in sensitivity between the two groups is therefore slightly underestimated.

Our group of breast cancer survivors is older than the group of women with no previous breast cancer diagnoses. As younger women are more likely to have dense breasts and mammography works best in fatty breasts, the found difference in sensitivity between these two groups is probably underestimated.

Most women who go for regular follow‐up outside the screening programme will have a clinical breast examination and/or a diagnostic mammography 0‐2 years after the screening. Breast cancers detected at this clinical breast examination or mammography will according to our definitions be classified as interval cancers, wherefore the sensitivity will be lower. We do not know whether a woman is going for regular follow‐up outside the screening programme. We do though know whether or not a woman had a diagnostic breast imaging 0‐21 months prior to the screening, which would be the case for most women going for regular follow‐up. As we found a significant lower sensitivity both for women with and without a diagnostic breast imaging 0‐21 months prior to the screening, it seems unlikely that the lower sensitivity among breast cancer survivors can be explained by their participation in regular follow‐up. The fact that we also found a significant lower sensitivity among women who had their latest breast cancer more than 10 years ago further strengthens this, since women have never previously been recommended follow‐up for more than 10 years.

### 4.2 Previous studies

Lee et al looked at risk of interval cancers up to 5 years after primary breast cancer in a cohort of breast cancer survivors diagnosed with DCIS or stage 0‐II unilateral breast cancer in the Breast Cancer Surveillance Consortium (US).^27^ Based on their reported numbers of screen‐detected cases and interval cancers their sensitivity was 73.7% among breast cancer survivors aged 50‐69 years. In another study of breast cancer survivors diagnosed with DCIS or stage 0‐II breast cancer in the Breast Cancer Surveillance Consortium, Houssami et al looked at screen‐detected and interval cancers up to more than 10 years after the previous breast cancer diagnosis for women of any age.^28^ They also only looked at interval cancers occurring up to 12 months after the mammography screening, and found that the proportion of screen‐detected cancers out of all cancers (SD/(IC + SD) was 65.4% for breast cancer survivors and 76.5 for women without previous breast cancer diagnoses. Both studies estimated sensitivity among breast cancer survivors to be quite higher than our estimated sensitivity, as these studies only included interval cancers occurring up to 12 months after mammography screening. If we only included interval cancers occurring up to 12 months after mammography screening our estimated sensitivity for breast cancer survivors would be 72.2% for initial screenings and 67.9% for subsequent screenings.

In a study from the Netherlands Lu et al looked at 5495 patients of any age with primary ipsilateral breast cancer diagnosed in four hospitals in the North Netherland.^29^ They estimated sensitivity to be 59.6% among all patients. This is a bit higher than our sensitivity as they only included interval cancers occurring before the next scheduled follow‐up appointment.

The study by Houssami et al was the only study that reported data from which it was possible to calculate the false positive risk.^28^ The false positive risk in the Houssami et al study was 1.7% among breast cancer survivors (with a previous DCIS or stage I + II cancer) and 1.0% among women without a previous breast cancer. This is different from our findings, showing that breast cancer survivors had a smaller or similar false positive risk compared to women without a previous breast cancer. The discrepancy might be due to Houssami et al only including women who previously had a DCIS or Stage I and II cancer or the fact that the data in the study by Houssami et al come from older screenings done between 1996 and 2007, where eg, digital mammography was not that common.

As also stated in the article by Houssami et al there can be several reasons for the lower sensitivity among breast cancer survivors compared to women without previous breast cancer diagnoses.^28^ Breast cancer survivors might have a larger risk of getting breast cancers that are less likely to be detected with mammography screening. Secondly, breast cancer survivors might be more aware and seek help promptly when experiencing breast symptoms and thirdly some interval cancers may be due to adjunct follow‐up occurring in between mammography screenings. Our study showed that breast cancer survivors have 30%‐40% less of their breast cancers detected at screening mammography compared to women without previous breast cancer diagnoses. This was similar both for women who had a diagnostic breast imaging 0‐21 months prior to mammography screening as well as among those who did not have such an examination. It therefore does not seem likely that the difference between breast cancer survivors and women without previous breast cancer diagnoses can be explained by whether or not they had adjunct follow‐up in between mammography screenings. It neither seems likely that more breast awareness can explain the difference.

In a recent study from South Korea of 188 breast cancer survivors aged 21‐79 years with a recurrence and a surveillance mammography within 1 year before the recurrence, Yeom et al reported that among the cancers that were not detected by mammography, 43% were due to cancer being obscured by dense breast tissue and 16% were due to the cancer being obscured by postoperative scar.^30^ Although our population of breast cancer survivors is older than the Yeom et al study population, it still seems likely that some of the lower sensitivity in our group of breast cancer survivors can also be explained by cancers being obscured by postoperative or irradiation scars. As we do not have data on breast density, we do not know how much of the lower sensitivity among breast cancer survivors can be explained by higher breast density among breast cancer survivors. If higher breast density among breast cancer survivors explains part of the lower sensitivity among breast cancer survivors then tomosynthesis or ultrasound could be valuable examinations.^31^

## 5 CONCLUSION

This study showed that breast cancer survivors have 30%‐40% less of their breast cancers discovered by mammography screening compared to women without previous breast cancer diagnoses. The lower sensitivity does not seem to be due to different behavior among breast cancer survivors, but some of it can probably be explained by cancers being obscured by postoperative or irradiation scars. To achieve a reasonable sensitivity among breast cancer survivors a lower screening interval or other examinations should be investigated.

## 6 ETHICAL REQUIREMENTS

The study is entirely based on register data, and no contact was made to patients, their relatives or treating physicians. According to Danish legislation, data from these registers can be used for research purposes after approval of the Danish Data Inspection Agency, without consent from individual citizens or approval from an ethical review board. This project was approved by the Danish Data Protection Agency (J. no.: 2012‐58‐0006/1‐16‐02‐89‐17.

## 7 AUTHOR CONTRIBUTION STATEMENT

Conception and design of the study: SHN, IV; Acquisition of data: SHN, IV; Analysis and interpretation of data: SHN, ML; Drafting of the manuscript: SHN; Revising manuscript for important content: IV, ML; Final approval of the submitted version: SHN, IV, ML.

## CONFLICT OF INTEREST

All authors declare no potential conflicts of interest.

## Data Availability

The data that support the findings of this study are available from The Danish Health Data Authority and The Danish Clinical Quality Program– National Clinical Registries (RKKP). Restrictions apply to the availability of these data, which were used under license for this study. Data may be available upon reasonable request to The Danish Health Data Authority and The Danish Clinical Quality Program – National Clinical Registries (RKKP).

## References

[1] Colleoni M , Sun Z , Price KN , et al. Annual hazard rates of recurrence for breast cancer during 24 years of follow‐up: results from the International Breast Cancer Study Group trials I to V. J Clin Oncol. 2016;34(9):927‐935. 26786933 10.1200/JCO.2015.62.3504 PMC4933127

[2] Kurtz JM , Amalric R , Brandone H , et al. Local recurrence after breast conserving surgery and radiotherapy; frequency, time course, and prognosis. Cancer. 1989;63:1912‐1917. 2702564 10.1002/1097-0142(19890515)63:10<1912::aid-cncr2820631007>3.0.co;2-y

[3] Lin NU , Thomssen C , Cardoso F , et al. International guidelines for management of metastatic breast cancer (MBC) from the European School of Oncology (ESO)‐MBC Task Force: Surveillance, staging, and evaluation of patients with early‐stage and metastatic breast cancer. Breast. 2013;22:203‐210. 23601761 10.1016/j.breast.2013.03.006

[4] Independent UK Panel on Breast Cancer Screening . The benefits and harms of breast cancer screening: an independent review. Lancet. 2012;380:1778‐1786. 23117178 10.1016/S0140-6736(12)61611-0

[5] Lauby‐Secretan B , Scoccianti C , Loomis D , et al. Breast‐cancer Screening — viewpoint of the IARC working group. N Engl J Med. 2015;372:2353‐2358. 26039523 10.1056/NEJMsr1504363

[6] Houssami N , Ciatto S . Mammographic surveillance in women with a personal history of breast cancer: how accurate? How effective? Breast. 2010;19:439‐445. 20547457 10.1016/j.breast.2010.05.010

[7] Taggart F , Donnelly P , Dunn J . Options for early breast cancer follow‐up in primary and secondary care ‐ a systematic review. BMC Cancer. 2012;12:238. 22695275 10.1186/1471-2407-12-238 PMC3502561

[8] Muradali D , Kennedy EB , Eisen A , Holloway CMB , Smith CR , Chiarelli AM . Breast screening for survivors of breast cancer: A systematic review. Prev Med. 2017;103:70‐75. 28765083 10.1016/j.ypmed.2017.07.026

[9] Holli K , Saaristo R , Isola J , Hyöty M , Hakama M . Effect of radiotherapy on the interpretation of routine follow‐up mammography after conservative breast surgery: a randomized study. Br J Cancer. 1998;78:542‐545. 9716041 10.1038/bjc.1998.529 PMC2063090

[10] Dershaw DD . Mammography in patients with breast cancer treated by breast conservation (lumpectomy with or without radiation). AJR Am J Roentgenol. 1995;164:309‐316. 7839960 10.2214/ajr.164.2.7839960

[11] van Breest Smallenburg V , Duijm LEM , Voogd AC , et al. Lower sensitivity of screening mammography after previous benign breast surgery. Int J Cancer. 2012;130:122‐128. 21328339 10.1002/ijc.25984

[12] van Breest SV , Duijm LE , Voogd AC , Jansen FH , Louwman MW . Mammographic changes resulting from benign breast surgery impair breast cancer detection at screening mammography. Eur J Cancer. 2012;48:2097‐2103. 22513229 10.1016/j.ejca.2012.03.011

[13] Bond M , Pavey T , Welch K , et al. Systematic review of the psychological consequences of false‐positive screening mammograms. Health Technol Assess. 2013;17(1–170):v‐vi. 10.3310/hta17130 PMC4781086 23540978

[14] Bolejko A , Hagell P , Wann‐Hansson C , Zackrisson S . Prevalence, long‐term development, and predictors of psychosocial consequences of false‐positive mammography among women attending population‐based screening. Cancer Epidemiol Biomarkers Prev. 2015;24:1388‐1397. 26311562 10.1158/1055-9965.EPI-15-0060

[15] København:Sundhedsstyrelsen . Sundhedsstyrelsen: Opfølgningsprogram for brystkræft [Follow‐up programme for breast cancer]. 2015; (in Danish).

[16] http://www.dbcg.dk/PDF%20Filer/Kap_9_Opfoelgning_og_kontrol‐11.12.2015.pdf (2015 guidelines on follow‐up of breast cancer patients from the Danish Breast Cancer Group) [Accessed April 4, 2019].

[17] http://dbcg.dk/PDF%20Filer/Retningslinier‐2004.pdf (2004 guidelines on follow‐up of breast cancer patients from the Danish Breast Cancer Group – in Danish)[Accessed April 4, 2019].

[18] http://www.dbcg.dk/PDF%20Filer/Gamle/Retningslinier%202010%20Kap%209%20%20010410.pdf ((2010 guidelines on follow‐up of breast cancer patients from the Danish Breast Cancer Group – in Danish)[Accessed April 4, 2019].

[19] http://www.dbcg.dk/PDF%20Filer/Kap%209%20Opfoelgning%20og%20kontrol_11.6.2012.pdf (2012 guidelines on follow‐up of breast cancer patients from the Danish Breast Cancer Group – in Danish)[Accessed April 4, 2019].

[20] http://web.drs.dk/kliniske‐retningslinier‐for‐mammografiscrenning‐i‐danmark‐revideret‐august‐2018/ (National guidelines on mammography screening – in Danish)[Accessed May 5, 2020].

[21] Vejborg I , Mikkelsen E , Garne JP , et al. Mammography screening in Denmark. Dan Med Bull. 2011;58(6):C4287. 21651881

[22] Fanizzi A , Basile TMA , Losurdo L , et al. A machine learning approach on multiscale texture analysis for breast microcalcification diagnosis. BMC Bioinformatics. 2020;21(Suppl 2):91. 32164532 10.1186/s12859-020-3358-4 PMC7069158

[23] Gjerstorff ML . the danish cancer registry. Scand J Public Health. 2011;39:42‐45. 21775350 10.1177/1403494810393562

[24] Mikkelsen EM , Njor SH , Vejborg I . Danish quality database for mammography screening. Clin Epidemiol. 2016;8:661‐666. 27822113 10.2147/CLEP.S99467 PMC5094619

[25] Lynge E , Sandegaard JL , Rebolj M . The danish national patient register. Scand J Public Health. 2011;39(7 suppl):30‐33. 21775347 10.1177/1403494811401482

[26] Bjerregaard B , Larsen OB . The danish pathology register. Scand J Public Health. 2011;39(7 Suppl):72‐74. 21775357 10.1177/1403494810393563

[27] Lee JM , Buist D , Houssami N , et al. Five‐year risk of Interval‐invasive second breast cancer. JnatlCancerInst (JNCI). 2015;107(7):djv109. 25904721 10.1093/jnci/djv109 PMC4651041

[28] Houssami N , Abraham LA , Migliorette DL , et al. Accuracy and outcomes of screening mammography in women with a personal history of early‐stage breast cancer. JAMA. 2011;305(8):790‐799. 21343578 10.1001/jama.2011.188 PMC3799940

[29] Lu W , Schaapveld M , Jansen L , et al. The value of surveillance mammography of the contralateral breast in patients with a history of breast cancer. Eur J Cancer. 2009;45(17):3000‐3007. 19744851 10.1016/j.ejca.2009.08.007

[30] Yeom YK , Chae EY , Kim HH , Cha JH , Shin HJ , Choi WJ . Screening mammography for second breast cancers in women with history of early‐stage breast cancer: factors and causes associated with non‐detection. BMC Med Imaging. 2019;19(1):2. 30611228 10.1186/s12880-018-0303-3 PMC6321714

[31] Tagliafico AS , Mariscotti G , Valdora F , et al. A prospective comparative trial of adjunct screening with tomosynthesis or ultrasound in women with mammography‐negative dense breasts (ASTOUND‐2). Eur J Cancer. 2018;104:39‐46. 30316869 10.1016/j.ejca.2018.08.029

